# Projecting Invasion Risk of Non-Native Watersnakes (*Nerodia fasciata* and *Nerodia sipedon*) in the Western United States

**DOI:** 10.1371/journal.pone.0100277

**Published:** 2014-06-25

**Authors:** Jonathan P. Rose, Brian D. Todd

**Affiliations:** 1 Department of Wildlife, Fish & Conservation Biology, University of California Davis, Davis, California, United States of America; 2 Graduate Group in Ecology, University of California Davis, Davis, California, United States of America; Universität Zurich, Switzerland

## Abstract

Species distribution models (SDMs) are increasingly used to project the potential distribution of introduced species outside their native range. Such studies rarely explicitly evaluate potential conflicts with native species should the range of introduced species expand. Two snake species native to eastern North America, *Nerodia fasciata* and *Nerodia sipedon*, have been introduced to California where they represent a new stressor to declining native amphibians, fish, and reptiles. To project the potential distributions of these non-native watersnakes in western North America, we built ensemble SDMs using MaxEnt, Boosted Regression Trees, and Random Forests and habitat and climatic variables. We then compared the overlap between the projected distribution of invasive watersnakes and the distributions of imperiled native amphibians, fish, and reptiles that can serve as prey or competitors for the invaders, to estimate the risk to native species posed by non-native watersnakes. Large areas of western North America were projected to be climatically suitable for both species of *Nerodia* according to our ensemble SDMs, including much of central California. The potential distributions of both *N. fasciata* and *N. sipedon* overlap extensively with the federally threatened Giant Gartersnake, *Thamnophis gigas*, which inhabits a similar ecological niche. *N. fasciata* also poses risk to the federally threatened California Tiger Salamander, *Ambystoma californiense*, whereas *N. sipedon* poses risk to some amphibians of conservation concern, including the Foothill Yellow-legged Frog, *Rana boylii*. We conclude that non-native watersnakes in California can likely inhabit ranges of several native species of conservation concern that are expected to suffer as prey or competing species for these invaders. Action should be taken now to eradicate or control these invasions before detrimental impacts on native species are widespread. Our methods can be applied broadly to quantify the risk posed by incipient invasions to native biodiversity.

## Introduction

Invasive species are a leading threat to native biodiversity [1], [2]. Their impacts include extinction of native species [3], shifts in trophic dynamics [4], and alteration of ecosystem processes [5]. An analysis of the extinctions caused by invasive species suggests that it is their role as predators through which they most often pose the greatest risk to native fauna [6]. In this regard, snakes, because they are universally predatory carnivores, are capable of having extensive impacts on ecological communities through predation on native species. For example, since its introduction to Guam, the Brown Tree Snake, *Boiga irregularis*, has nearly eliminated the island's native bird and lizard fauna [7], [8], with cascading effects on other native taxa [9]. A recent study has also shown a remarkable decline in the abundance of native mammals in Everglades National Park that coincided with the introduction and wider establishment of the Burmese Python, *Python molurus bivittatus* [10]. These cautionary examples suggest that snakes as a group deserve special attention when screening potential invaders and monitoring recently established non-native species. This is especially true for habitats already subject to degradation from other anthropogenic activities because the density of invasive species is often elevated in altered communities [11].

Western watersheds in North America have been besieged by invasive species that have capitalized on ecosystem-level alterations such as water diversion and damming that have changed flow regimes substantially [12], [13]. Consequently, native fish and amphibians have undergone significant declines in much of the west [14], [15], with invasive species identified as important contributors to these declines [14], [16]. The invasion of western North America's ecosystems by non-native species is highlighted by the recent establishment of two snake species of the genus *Nerodia*. The Southern Watersnake, *Nerodia fasciata*, native to the southeastern USA, was first reported in in Los Angeles, California (CA), USA in 1976, and reproductive populations were first documented in Folsom, CA in 1992 and in Harbor City, CA in 2006 [17]–[19]. The Common Watersnake, *Nerodia sipedon*, which ranges across much of eastern North America, was first documented in Roseville, CA in 2007 [20]. Subsequent work in Roseville has shown that there is a dense, reproductive population of *N. sipedon* located just 13 km from populations of the federally threatened Giant Gartersnake, *Thamnophis gigas* [21], with which it shares a similar ecological niche. To date, there has been no effort to quantify the risk of *Nerodia* becoming more widely established in western North America, a significant possibility given the highly networked, anthropogenically-modified freshwater systems in this region.

A crucial first step in evaluating the risk posed by an incipient invader is to model its potential distribution within the invaded range. Climate matching has proven to be a powerful predictor of establishment probability for reptiles and amphibians [22], and models based on the climatic niche of a species in its native range can accurately predict invasive range [23], [24]. Still, there can be uncertainty in a species' potential distribution due to differences in modeling methods or the predictor variables used to build the model [25], [26]. This uncertainty can be incorporated into the modeling process by using an ensemble approach where the results of multiple models are integrated to present a range of possible invasion scenarios [27]. While the ensemble approach is a promising step forward in risk analysis for invasive species, most modeling efforts still fail to make the connection to potential impacts on native species. If there is abundant suitable habitat for an invasive species, the next step is to evaluate the potential for the invader to co-occur with, and therefore potentially interact with, native species. In the case of introduced watersnakes, they may be expected to interact with a wide range of native species due to their generalist diets and broad habitat preferences [28].

Here we present Species Distribution Models (SDMs) for *N. fasciata* and *N. sipedon* and quantify the risk posed by these non-native watersnakes to native communities in western North America. Our study had two main objectives: 1) to project the potential distribution of *N. fasciata* and *N. sipedon* in western North America, and 2) to calculate the risk posed by these invaders to an assemblage of native species in California. We use an ensemble modeling approach with three machine learning algorithms to predict the potential invasive range of these species. We evaluate the transferability of our models to new environments using a rigorous, spatially-stratified cross-validation method. We then combine knowledge of the ecology and life-history of the invaders with a spatial analysis of native biodiversity to estimate risk to imperiled native fauna. This type of analysis can highlight regions where non-native species are expected to invade and help determine whether and where these species deserve attention from state or federal agencies based on range overlap with imperiled native species for which they pose a conservation risk. The results of our study have explicit management implications for the two introduced species we examined. However, our methods can also be more broadly applied to understanding the threat posed to native species by other potential or incipient invaders.

## Materials and Methods

### Species occurrences and environmental data

We downloaded species occurrence records from the Global Biodiversity Information Facility (http://data.gbif.org) and HerpNET (http://herpnet.org/portal.html) online databases, with additional records from the Carolina Herp Atlas [29] and the Tennessee Herp Atlas (A. Floyd Scott, pers. comm.). We georeferenced occurrences using locality information associated with specimen records and only kept records with an estimated precision of <5 km according to MaNIS standards [30]. To minimize the confounding impact of spatial autocorrelation on modeling results [31], [32], we used a 10 arc-minute raster to spatially filter occurrences such that each cell contained only one occurrence record [33]. After spatial filtering, we were left with 1,067 records for *N. sipedon* and 460 records for *N. fasciata*. We included occurrences of *N. fasciata* and *N. sipedon* from California in addition to occurrences from the native range when building models because including both has been shown to improve predictive performance of SDMs [34].

The selection of which environmental variables to use to characterize a species' niche is a critical step when creating SDMs, and poor choice of predictors can lead to under-prediction of a species' invasive range [24], [25]. False negatives (incorrectly predicting absence at a known presence) are more costly than false positives (incorrectly predicting presence where it is absent) for SDMs in general [35], and especially so for invasive species applications [36]. Therefore, it is important to avoid over-fitting a model to a species' native range by including many, collinear variables when projecting the potential distribution of invasive species [37]. We sought to avoid this problem using an Ecological Niche Factor Analysis (ENFA; [38]) to identify which climatic variables had the greatest influence on the native distributions of *N. fasciata* and *N. sipedon*. We limited our analysis to the 11 temperature variables from the WorldClim dataset [39] because temperature has direct physiological effects on ectotherms such as reptiles [40], and models based on such “direct gradients” are more generalizable [41]. The results of the ENFA identified three variables for use in the SDMs that each had a large influence in determining the distributions of our study species and that were not highly correlated with one another: temperature seasonality (Bio 4), mean temperature of the warmest quarter (Bio 10), and mean temperature of the coldest quarter (Bio 11).

Appropriate temperatures alone are not sufficient to make an area suitable for watersnakes; wetland habitats must also be present for these species to persist. We did not use the precipitation variables in the WorldClim dataset because precipitation does not necessarily indicate the availability of wetland habitat. For example, the known occurrences of *N. fasciata* in California receive less than 65% of the annual rainfall of the driest occurrence in this species' native range, and almost no rainfall during the summer, yet these populations are well established and adequate wetland habitat is widespread [20]. In other words, low precipitation does not necessarily mean an absence of suitable wetland or other aquatic habitat on which these watersnakes depend. To remedy this problem, instead of using precipitation, we created a raster layer of wetland habitat using data from the USGS National Hydrography Dataset (NHD) (http://nhd.usgs.gov/data.html) and the Canadian Wetlands Inventory from the Ontario Ministry of Natural Resources. Both of these datasets represent surface water resources using polygons, and they classify water bodies depending on their origin, permanence, and flow (e.g., lentic vs. lotic). Based on the habitat preferences of *N. fasciata* and *N. sipedon*, we selected water bodies from these GIS datasets that were classified as perennial natural and artificial streams, lakes/ponds, reservoirs, and swamps/marshes. We removed desert playas, saline lakes, estuaries, peat bogs, and intermittent streams and washes from the dataset as these water bodies are not suitable for watersnakes. We then created a 2.5 arc minute grid (matching the resolution of the WorldClim data) covering the contiguous US and Ontario, and calculated the total area of wetland resources within each cell using ArcGIS version 9.3 (ESRI, Redlands, CA). The final product is a raster layer covering our study area with an estimate of the proportion of land cover that is wetland habitat in 2.5 arc minute cells across the contiguous US and Ontario. We then used this “wetland” layer as the fourth predictor variable in our SDMs (see Appendix S1).

### Species Distribution Modeling

We created models with three widely used algorithms that have been shown to perform well at estimating species' distributions from occurrence records: Boosted Regression Trees [42], Random Forests [43], and Maxent [44]. Maxent is a machine learning method designed to be used with presence-only data. Maxent estimates a probability density function that best fits the environmental characteristics of species occurrence data while being minimally different from a probability density function describing the environmental characteristics of the entire study region [45]. Maxent has been widely used to model potential distributions of invasive species [23], [24], [46] and has outperformed other niche modeling methods in direct comparisons [47], [48]. For Maxent, we selected logistic output, and set the model to clamp values to the maximum value in the training data set when projecting outside the range of the training data. We combined the presences for each species with the pseudo-absences described below to generate background points for Maxent.

Boosted Regression Trees (BRT) is a machine learning technique that combines many simple regression trees with one or a few nodes into one model with greater predictive ability [49], and has proven effective at modeling species' distributions [47], [50]. Random Forests (RF) is also based on regression trees and combines the predictions of many trees, each built using a bootstrapped sample of the data [51]. When building each node of a tree, Random Forests randomly selects only a subset of the environmental predictors for binary partitioning, which limits bias [52]. See Appendix S1 for details on parameter settings for the three modeling methods. We generated “pseudo-absences” equal in number to the known presences for each species, at least 2° from known presence points following [53]. Pseudo-absences were constrained to be within the geographical area “accessible” to *Nerodia* following the recommendations of Barve et al. [54] (see Appendix S1 for details on background selection).

Projecting the potential distribution of an invasive species involves estimating suitability in regions in which the model has not been trained. To evaluate how well our models perform at extrapolating beyond their training area, we partitioned occurrence points into separate training or test subsets using five latitudinal bands, and performed a *k*-fold cross-validation on the five subsets (see Appendix S1). This type of spatially-stratified validation is a more robust evaluation method than randomly withheld testing points if the goal of the model is to extrapolate into areas outside of the boundaries of the training data [36], [55]. We used area under the receiver operating characteristic curve (AUC) on withheld data to evaluate model predictive capabilities. While AUC has shortcomings as a model evaluation statistic, such as its dependence on the geographical extent of the background used to construct the model [56], it is valid here because we use it to compare models for the same species with the same background that differ only in the machine learning technique used to build the model. After model evaluation, we ran one final model for each set of climatic variables with all occurrences (including those from the invasive range) used as input to maximize the amount of data available for model construction.

To create an ensemble prediction of climatic suitability that combines the output from the three modeling algorithms, we calculated a simple average of the three models' output. We intended to use a weighted average where each models prediction was weighted by its AUC in our spatially stratified validation, such that better performing models' received greater weight. However, because of the substantial congruence in performance from the three models (see results below), weighting did not materially affect the ensemble model and we chose a simple mean of the three models' predictions instead. As another form of model evaluation, we calculated the omission error rate for our ensemble models, i.e. the number of presence records incorrectly classified as absent. To convert the continuous model output to a binary presence-absence prediction, we used the maximum of the sum of sensitivity and specificity as a threshold (see below for details on this threshold choice).

### Overlap with native species

We evaluated the overlap between the ranges of California's native Gartersnakes (genus *Thamnophis*) and the potential distribution of *N. fasciata* and *N. sipedon* because these species are closely related [57], and inhabit similar ecological niches. *N. fasciata* and *N. sipedon* are generalist predators of fish and amphibians, and share similar diets and life-histories with many snakes of the genus *Thamnophis*. We estimated overlap for the two *Nerodia* species with all members of the genus *Thamnophis* native to California, except *Thamnophis marcianus*, which primarily inhabits arid regions, and *T. sirtalis*, because it is a habitat generalist and the only species in our study region with which *N. fasciata* and *N. sipedon* can co-occur in their native range. Note we do include the San Francisco Gartersnake, *Thamnophis sirtalis tetrataenia*, a subspecies of *T. sirtalis*, in our analysis because the San Francisco Gartersnake specializes on aquatic habitats such as ponds and marshes where it feeds mainly on amphibians [58] and therefore is more likely to compete with introduced watersnakes.

We also identified native fish and amphibian species expected to serve as prey and be negatively affected by the establishment of *N. fasciata* and *N. sipedon* based on the following criteria:

1. The species is of conservation concern at the state or federal level.
2. The species is predated upon by *N. fasciata* and/or *N. sipedon* in the snakes' native ranges (e.g., Rainbow Trout, *Oncorhynchus mykiss*).
3. The species is in the same genus or family as a fish or amphibian predated upon by *N. fasciata* and/or *N. sipedon* in the snakes' native ranges (e.g., *Ambystoma californiense*, *Rana draytonii*, *Deltistes luxatus*).
4. The species' body size, habitat preferences, and life-history make it likely prey for generalist aquatic predators such as *N. fasciata* and *N. sipedon* (e.g., *Dicamptodon ensatus*, *Hysterocarpus traskii*).

In order to be selected, a species had to meet criterion 2, or meet both criterion 1 and one of criteria 3 or 4.

We then compared the known distributions of these native species to the potential distributions of introduced *N. fasciata* and *N. sipedon* to estimate their potential for co-occurrence. We used range maps for native species (see Table S1) and our ensemble model outputs for the introduced watersnakes to calculate the suitability of each native species' range for *N. fasciata* and *N. sipedon*. To estimate the proportion of the native species' range that is suitable for introduced *Nerodia*, we first had to convert our continuous ensemble model output to a binary presence/absence output using a threshold. We chose the threshold that maximized the sum of the sensitivity and specificity (Max SSS) of the model, a method that is valid for the presence-pseudo-absence data used in this study [59], and which has proven effective at classifying independent test data [60]. The proportion of a native species' range that is suitable for one of the introduced watersnakes gives a coarse measurement of the risk each native species faces should these introduced snakes become more widely established throughout California and western North America to those areas projected to be environmentally suitable.

### Connectivity to established populations

To determine the connectivity between established non-native populations and potentially suitable habitat for *N. fasciata* and *N. sipedon*, we used a modified cost distance method implemented in ArcGIS. We first classified all cells as either suitable or unsuitable using the Max SSS threshold as described above. We then calculated for each suitable cell whether or not it was connected to an established non-native watersnake population using the “Cost Distance” tool in ArcMap. The established non-native population(s) of a species acted as the source, and movement was only allowed through suitable cells, with unsuitable cells acting as barriers. This produced a map that identifies areas that are not only environmentally suitable for introduced *Nerodia* according to our models, but also are connected to source populations such that we would expect watersnakes to be able to reach them through natural dispersal, without added human intervention.

## Results

The distribution of *N. fasciata* was most influenced by the mean temperature of the warmest (Bio 10) and coldest (Bio 11) quarters according to the BRT and Maxent models. Temperature seasonality (Bio 4) and availability of wetland habitat (wetland) had little influence on *N. fasciata* occurrence according to BRT and Maxent models. The RF model differed in selecting wetland habitat as the most important variable, according to the increase in prediction error when observed values for this variable are randomly permuted. Bio 11 was the second most important variable for *N. fasciata* according to RF, followed by Bio 10, and then Bio 4. All three modeling methods produced similar response curves for *N. fasciata* (Figures S1–S3). Bio 10 displays a unimodal response, with highest suitability at temperatures ranging from 24–28°C, and suitability declining more sharply at temperatures below 24°C than temperatures above 28°C. For Bio 11, suitability increases sharply once temperatures increase above 0°C, before plateauing for temperatures above 10°C. *N. fasciata* shows a sharp linear response to wetland habitat availability, with suitability increasing linearly with proportion of wetland habitat in a cell from 0 to about 0.05 (0.2 for Maxent), and plateauing for values above this threshold. Finally, suitability for *N. fasciata* declines as temperature seasonality (Bio 4) increases for all three models, with Maxent exhibiting a sharp threshold response, whereas BRT and RF fit more gradually declining functions.

The suitability of an area for *N. sipedon* is primarily driven by the presence of wetland habitat, followed by Bio 10, then Bio 11 (Bio 4), and Bio 4 (Bio 11) according to our BRT (RF) model. In contrast, Maxent identifies Bio 4 as the variable making the greatest contribution to the model, followed by nearly equal contributions from wetland and Bio 10, with Bio 11 having only a small effect on model output. The environmental response curves for *N. sipedon* are very similar for all three modeling methods. For both Bio 4 and Bio 10, the response is unimodal, with the highest suitability at intermediate values of temperature seasonality and at a mean temperature of the warmest quarter between approximately 16–27°C (Figures S4–S6). The response curve for Bio 11 differs among models, with the Random Forest model fitting a unimodal response, Maxent fitting a function where suitability slowly increases with temperature, and BRT a function where suitability increases with temperature before plateauing and slightly declining at temperatures above 0°C. The response curve for wetland availability is the same for all three models and closely follows that for *N. fasciata* described above, a function that initially increases linearly, and then plateaus above 0.04 for BRT and RF or 0.2 for Maxent. The fact that the response curves for both species are not entirely smooth functions may raise concerns of over-fit models; however our response curves do not exhibit the sharp peaks and troughs indicative of over-fitting and are all intuitive given the biology of our study species.

Based on our spatially-stratified validation, all three models demonstrated excellent performance when projecting environmental suitability outside of the training range. For *N. fasciata*, the Maxent model had the highest mean AUC on withheld latitudinal bands (0.984, range 0.959–0.997) followed by the RF model (0.977, 0.932–0.999), and the BRT model (0.974, 0.929–0.993). The ranking of models was the same for *N. sipedon*; the Maxent model had the highest mean AUC (0.947, 0.902–0.996), followed by the RF model (0.943, 0.882–0.999), and the BRT model (0.933, 0.865–0.991). The model that performed best varied depending on the latitudinal band withheld for evaluation, and therefore no model was “best” at extrapolating beyond the training range in all cases (see Tables S2, S3). Also, all models performed slightly worse when the southernmost (Band 1) or northernmost (Band 5) training data was withheld. Our AUC values demonstrate that models created with different methods were similar in their predictive ability within the native range. Despite their similar performance according to AUC, these models give slightly different projections for the potential invasive range of *N. fasciata* and *N. sipedon* in western North America. For this reason, we present ensemble forecasts for the potential distribution of *N. fasciata* and *N. sipedon* that average the predictions of our three models.

**Figure 1:**
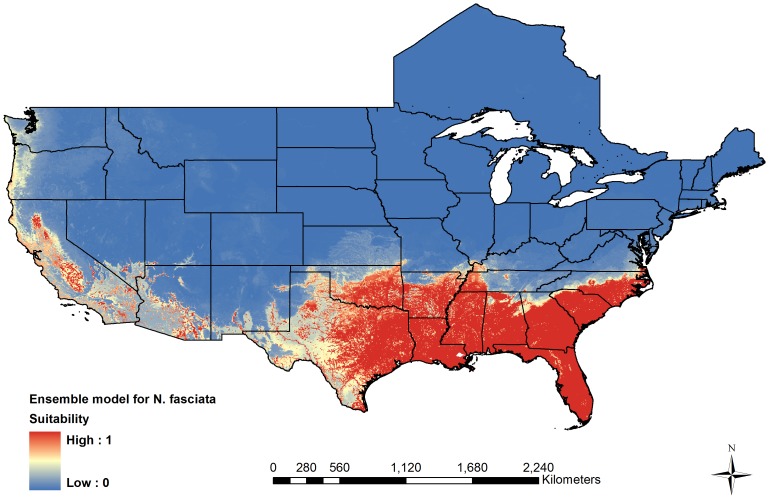
Projection of the potential distribution of the Southern Watersnake, *Nerodia fasciata*. Suitability from an ensemble model representing the average output from a MaxEnt, Boosted Regression Tree, and Random Forests model.

Figure 1 displays the ensemble forecast for *N. fasciata*. All three models agree in predicting large portions of California's Central Valley as suitable for *N. fasciata*, with all three models predicting suitable climate and sufficient wetlands in the northern portion of the valley near the currently established Folsom population. The ensemble model also predicts suitable climate and wetlands along the Pacific coast from central Oregon to southern California. The BRT and RF models both predict high suitability along the California coast and near the Los Angeles population of *N. fasciata*. However, the Maxent model predicts very low suitability along the Pacific coast of California and Oregon and low suitability for the *N. fasciata* population near Los Angeles. Our ensemble model also predicts some isolated areas with high suitability in southeastern California and southern Arizona and Nevada. This is due to the presence of surface waters such as Lake Mead and the Colorado River in otherwise dry areas that have sufficiently warm temperatures for *N. fasciata*. The ensemble model performs well at re-creating the native range of *N. fasciata*, and has a very low rate of omission error, with only 0.7% of presences predicted absent. The ensemble model displays some over-prediction into western Texas and northern Mississippi, Alabama, and Georgia, and this pattern is present in all three component models.

**Figure 2:**
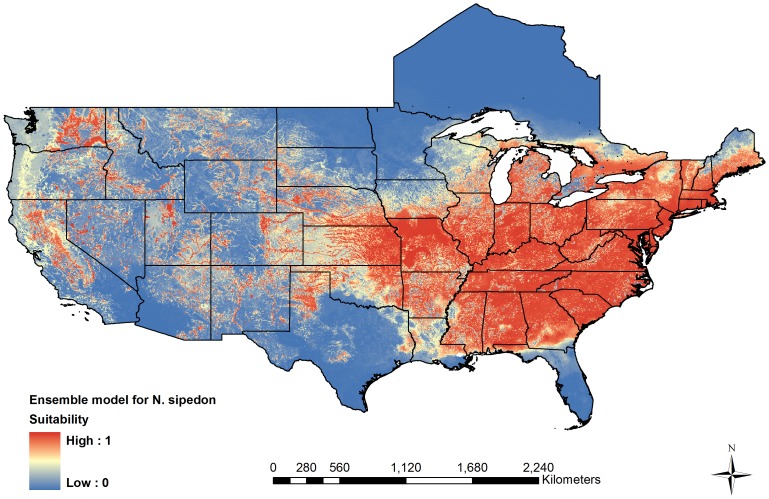
Projection of the potential distribution of the Common Watersnake, *Nerodia sipedon*. Suitability from an ensemble model representing the average output from a MaxEnt, Boosted Regression Tree, and Random Forests model.

Large swaths of western North America are climatically suitable for *N. sipedon* according to our ensemble model (Figure 2). Much of the Central Valley, foothills of the Sierra Nevada Mountains, and north-central California have high suitability values. The Pacific coast from San Francisco north to Washington is marginally suitable, with higher values at lower elevation in the Willamette Valley of Oregon. Additional areas in eastern Washington and southern Idaho have high suitability as well. Through much of the desert southwestern US, there are fragmented pockets of high suitability where lakes and permanent rivers occur. The ensemble model for *N. sipedon* also slightly over-predicts into areas that are unoccupied but adjacent to this species' native range, specifically in northern Texas, western Oklahoma, western South Dakota, and eastern Wyoming. The model shows some evidence of under-prediction on the northern edge of *N. sipedon*'s native range, with low suitability for eastern Minnesota and western Wisconsin. Consequently, the ensemble model for *N. sipedon* has a slightly higher omission error rate than seen for *N. fasciata*, with 3.1% of known presences predicted absent.

### Overlap with native species

**Figure 3:**
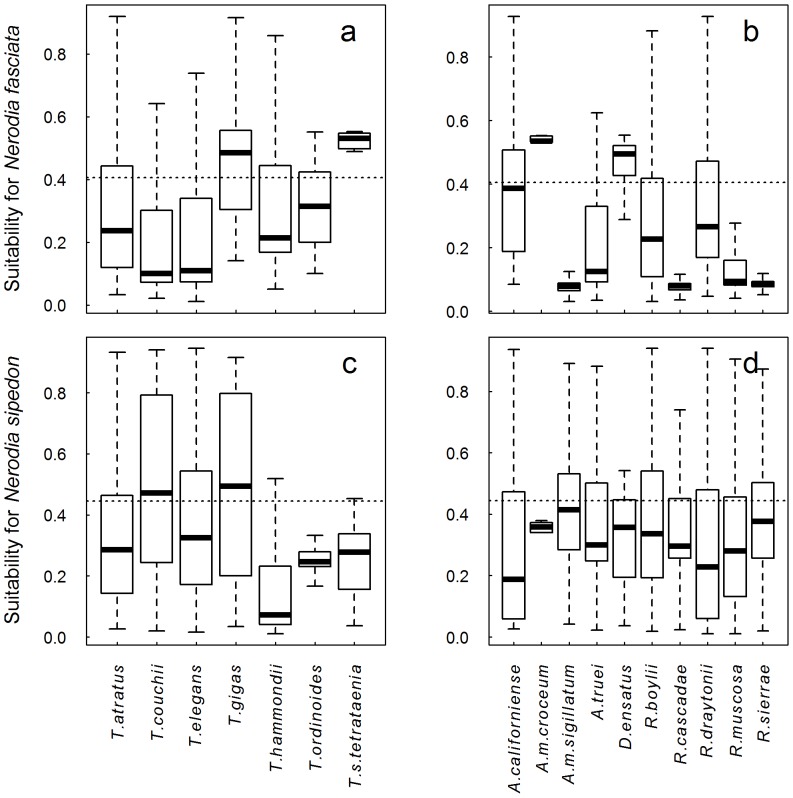
Range overlap between native Gartersnakes or amphibians and projected distribution of non-native watersnakes. Overlap with *N. fasciata* (a, b) and *N. sipedon* (c, d). The dotted horizontal line represents the maximum of the sum of sensitivity and specificity threshold; the larger the proportion of the boxplot above this line, the larger the proportion of the native species' range that is projected to be suitable for introduced watersnakes. The thick line in each boxplot represents the median suitability of each native species' range for the watersnake species, and the bottom and top of the box represent the first and third quartiles respectively. The whiskers extend to the most extreme data point that is within 1.5 times the interquartile range from the box.

**Figure 4:**
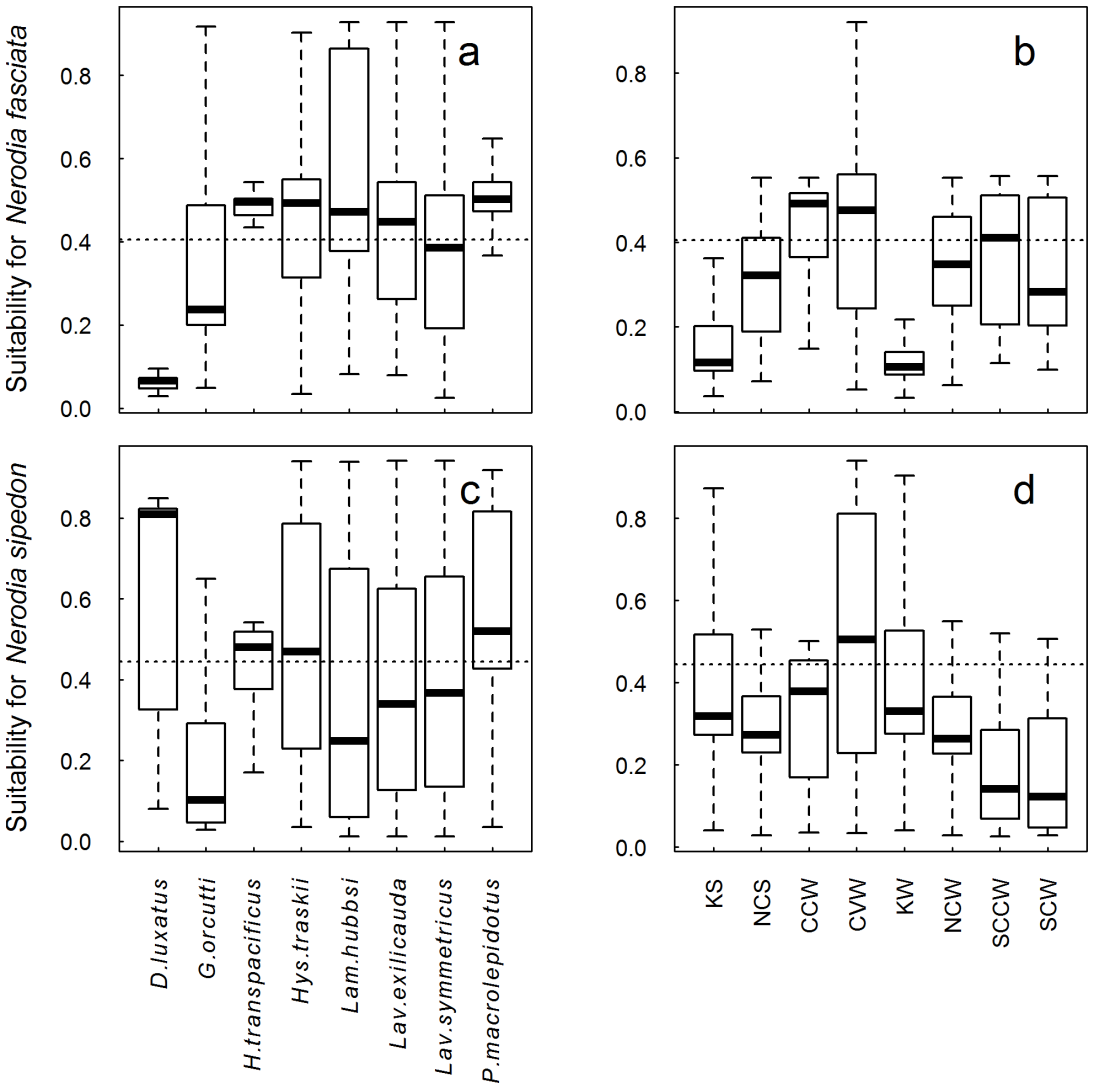
Range overlap between native fish and steelhead populations and projected distribution of non-native watersnakes. Overlap with *N. fasciata* (a, b) and *N. sipedon* (c, d). The dotted horizontal line represents the maximum of the sum of sensitivity and specificity threshold; the larger the proportion of the boxplot above this line, the larger the proportion of the native species' range that is projected to be suitable for introduced watersnakes. Abbreviations for steelhead populations are as follows: KS =  Klamath summer, NCS  =  North Coast summer, CCW  =  Central Coast winter, CVW  =  Central Valley winter, KW  =  Klamath winter, NCW  =  North Coast winter, SCCW  =  South Central Coast winter, SCW  =  South Coast winter. The thick line in each boxplot represents the median suitability of each native species' range for the watersnake species, and the bottom and top of the box represent the first and third quartiles respectively. The whiskers extend to the most extreme data point that is within 1.5 times the interquartile range from the box.

For each amphibian, fish, and reptile species native to California that are likely to interact with *N. fasciata* and *N. sipedon*, we calculated the suitability of all 2.5×2.5 arc minute cells within its range for *N. fasciata* and *N. sipedon* and present this information in the form of box and whisker plots (Figures 3 and 4). We also calculated the proportion of the native species' range that is above the Max SSS threshold. Comparing native snakes in the genus *Thamnophis*, *N. fasciata* has the potential to overlap greatest with the ranges of two species: the federally threatened Giant Gartersnake, *T. gigas* (69.5% of *T. gigas* range is predicted suitable for *N. fasciata*) and the federally endangered San Francisco Gartersnake, *Thamnophis sirtalis tetrataenia* (80.6%; Figure 3a). *Nerodia sipedon* also has the potential to overlap with a majority of the range of *T. gigas* (55.7%) and also shows substantial potential for overlap with the Sierra Gartersnake, *Thamnophis couchii* (54%; Figure 3c), a species that is not currently of conservation concern.

*Nerodia fasciata* has the potential to inhabit a substantial portion of the range of three native California amphibians, the Santa Cruz Long-toed Salamander, *Ambystoma macrodactylum croceum* (83.3%), the California Giant Salamander, *Dicamptodon ensatus* (76.5%), and the California Tiger Salamander, *Ambystoma californiense* (47.5%; Figure 3b). In contrast, *N. sipedon* is only predicted to overlap with large parts of the range of two amphibian taxa, the Southern Long-toed Salamander, *Ambystoma macrodactylum sigillatum* (44.4%), and the Foothill Yellow-legged Frog, *Rana boylii* (39.3%; Figure 3d).

For native fish species in California, greater than half the range of the Delta Smelt, *Hypomesus transpacificus* (83.2%), Tule Perch, *Hysterocarpus traskii* (69.2%), Kern Brook Lamprey, *Lampetra hubbsi* (62.2%), Hitch, *Lavinia exilicauda* (57.6%), and Sacramento Splittail *Pogonichthys macrolepidotus* (83.8%) is predicted suitable for *N. fasciata* (Figure 4a). *N. sipedon* overlaps substantially with the ranges of four other native fish species: the Lost River Sucker, *Deltistes luxatus* (71%), and *Hypomesus transpacificus* (68.2%), *Hysterocarpus traskii* (57.6%) and *P. macrolepidotus* (73.6%; Figure 4c).

Three steelhead *Oncorhynchus mykiss* populations show a high degree of overlap with *N. fasciata*, the Central Coast winter run (72.4%), the Central Valley winter run (64.1%) and the South Central Coast winter run (50.1%; Figure 4b). The potential distribution of *N. sipedon* only overlaps to a large degree with the range of Central Valley winter run steelhead (57.1%; Figure 4d).

### Connectivity to established populations

**Figure 5:**
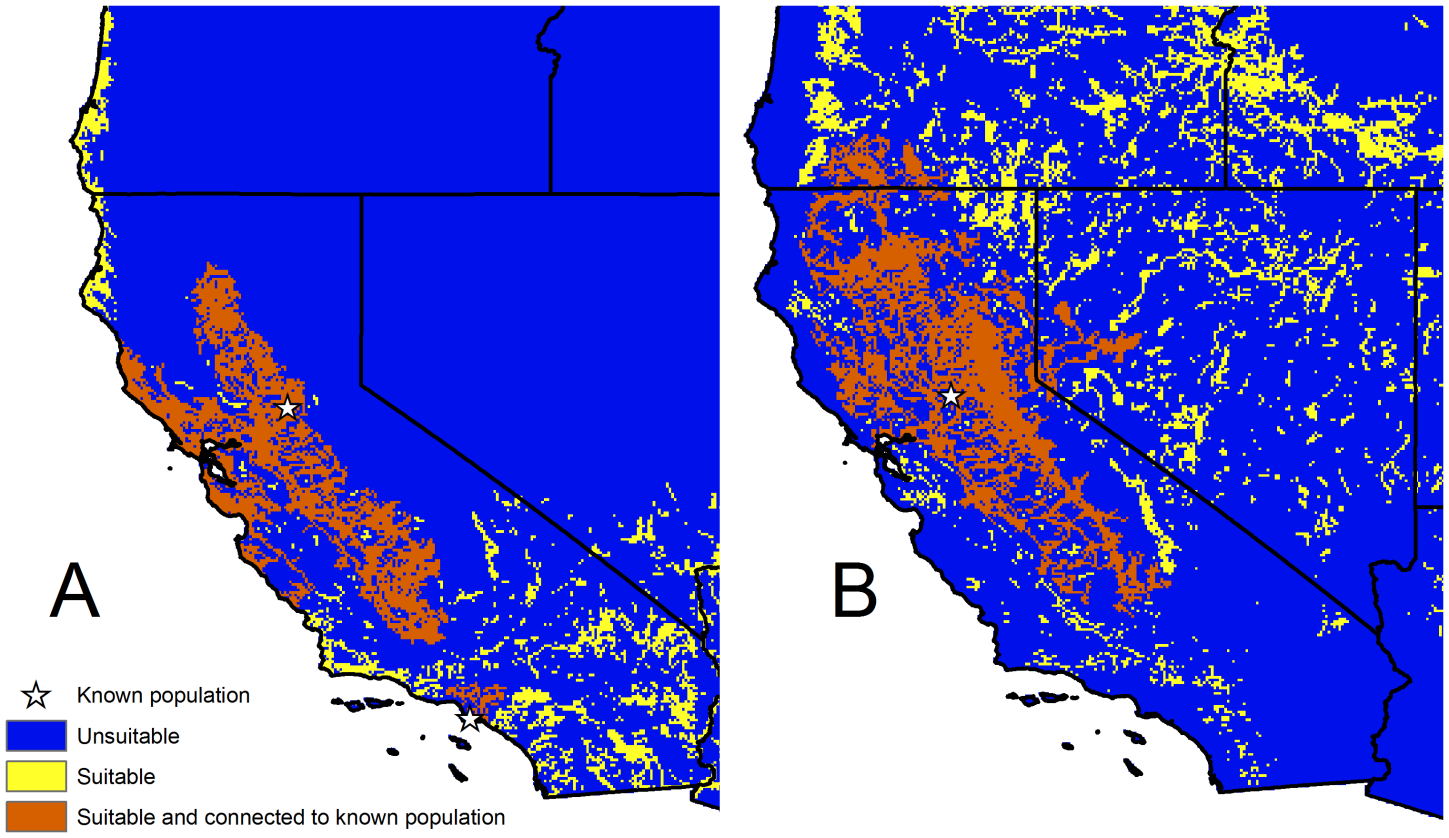
Connectivity of predicted suitable habitat to established non-native populations for non-native watersnakes. Connectivity to established populations for (A) *Nerodia fasciata* and (B) *Nerodia sipedon*. Classification into suitable and unsuitable areas was done using the maximum of the sum of sensitivity and specificity threshold for ensemble species distribution models.

The majority of California predicted to be environmentally suitable for *N. fasciata* and *N. sipedon* is also directly connected to currently established populations (Figure 5). For *N. sipedon*, the suitable regions in the Central Valley, Sierra Nevada foothills, and north-central California are connected to the population in Roseville, CA. Similarly for *N. fasciata*, the Central Valley and central coast regions are connected through suitable environments to the Folsom population, whereas the Harbor City population is more isolated, resulting in a smaller area being classified as both suitable and connected along the south coast. Indeed, areas that are environmentally suitable but not connected to known populations can be found in southern California for both species. We reanalyzed the potential overlap between native species' ranges and areas suitable for watersnakes using only those areas connected to established watersnake populations and found qualitatively similar results. For the species listed above whose ranges overlap extensively with areas suitable for non-native watersnakes, the percentage of overlap declined on average by just 1.7% (0–12.3%) for *N. fasciata* and 2.8% (0–7.5%) for *N. sipedon*.

## Discussion

Our results demonstrate that large areas of western North America are climatically suitable for *N. fasciata* and *N. sipedon* and that these suitable areas overlap considerably with the ranges of several already imperiled species native to California. For both introduced watersnake species, the distribution models predict much of California's Central Valley to be highly suitable. In other areas, the predictions differ for the two species, with cooler northern and higher elevation areas predicted suitable for the northerly distributed *N. sipedon*, whereas warmer southern, coastal, and lower elevation areas are predicted suitable for the southeasterly distributed *N. fasciata.* Due to these differences in climatic niche, the two *Nerodia* species pose threats to different native aquatic species should they expand beyond their current populations. For example, *N. fasciata* has the potential to inhabit large parts of the range of the federally endangered San Francisco Gartersnake, *T. s. tetrataenia* and Santa Cruz Long-toed Salamander, *A. m. croceum*, as well as threatened species such as the Giant Gartersnake, *T. gigas* and California Tiger Salamander, *A. californiense*, and the California Giant Salamander, *D. ensatus*, a state species of special concern (SSC). All of these imperiled species are distributed either in the Central Valley or along the central and southern California coast. *Nerodia sipedon*'s projected distribution also overlaps greatly with the Giant Gartersnake, but it appears more likely to conflict with California SSCs such as the Southern Long-toed Salamander, *A. m. sigllatum*, and the Foothill Yellow-legged Frog, *R. boylii*, than does *N. fasciata*. Both species of *Nerodia* could inhabit large portions of the range of several imperiled fish species native to the Central Valley, and *N. fasciata* has the potential to occur in spawning habitats used by threatened steelhead populations. While neither species of introduced watersnake currently co-occurs with these native species of conservation concern, *N. sipedon* has been documented feeding on native amphibians in California [61], and both watersnakes are generalist predators that will eat any aquatic prey of suitable size [28]. The wide range of imperiled species potentially threatened by these snakes, and the historical precedent of negative impacts on native communities by introduced snake species [7], [8], [10] provides a powerful incentive for management action that eradicates currently established populations before they spread further.

One of the greatest challenges for SDMs is projecting into spaces and times that were not used to build the model, often called model “transferability” [62]. This is a significant challenge when projecting the potential distribution of invasive species, as we are often forced to build models based primarily on occurrences from a species' native range and apply these models to a novel environmental space in the introduced range. We took two steps to ensure that our models would perform well at transferring to new environments. First, following the terminology of Austin [41], we only chose predictor variables that have a direct physiological effect on watersnakes, such as temperature, or are proximal predictors, such as the availability of aquatic habitat. Second, we used a spatially-stratified cross-validation method to evaluate our models' performance and constrain their complexity. Evaluating models based on their ability to predict independent, non-random spatial subsets of the data is a strong test of transferability and is necessary when attempting to make inferences beyond the region used to build the model [63]. While SDMs built with machine learning algorithms can become over-fit to the training data and perform poorly when transferring to new environments [46], [63], our spatially-stratified cross-validation allowed us to calibrate our models to produce smoother response curves that perform well when applied to novel environments. Our three models all performed well at predicting to novel, spatial subsets of occurrence data according to our model evaluation metric, which gives us confidence in our models' ability to project the potential distribution of watersnakes in western North America. The three modeling methods provided varying predictions of the potential distribution of *Nerodia* however, supporting the use of ensemble methods. For example, if we had only used Maxent to model the distributions of *N. fasciata* and *N. sipedon*, we would not have predicted parts of the Pacific coast to be suitable for these species. With an ensemble model, anomalous results are tempered and areas predicted as suitable by more than one model demonstrate greater support and more likely risk of establishment by *Nerodia*. Thus, using an ensemble approach may be especially important when projecting an SDM to a new region, as the inherent uncertainty of model transferability is better represented by the level of agreement between multiple component models than a single model output.

All three models produced response curves for each environmental predictor that were similar, despite the different algorithms used to model suitable environments (Figures S1-S6), bolstering confidence in our model results. Although the response curves display some complexity that likely reflects artifacts of occurrence data, for the most part they represent unimodal, sigmoidal, and negative exponential curves that are biologically plausible for these species. *Nerodia fasciata* and *N. sipedon* differ in intuitive ways, with suitability peaking at higher temperatures during the warmest and coldest parts of the year for *N. fasciata*, which has a more southerly distribution than *N. sipedon*, which ranges as far north as Ontario, Quebec, and Maine. The response curves also do not exhibit sharp changes near the edge of the sampled environment, which reduces concerns about extrapolating beyond the training range of the data [46]. An important, related point to note is that the fragmented nature of our ensemble species distribution models is not due to overly complex response curves (Figures S1–S6) and therefore does not indicate over-fit models. The fragmented nature of our model output is largely due to the spatial structure of our wetlands predictor variable, which varies much more at a fine spatial scale than the interpolated temperature variables from the WorldClim dataset. This can lead to sharp differences in predicted suitability between nearby cells if one has ample wetland habitat and the other does not. This explains why some cells within the native range of these watersnakes have low suitability scores according to our models: they do not have sufficient surface water available to provide habitat for these snakes.

While species distribution modeling using bioclimatic data can estimate whether broad areas are suitable for an invasive species, finer scale factors like habitat and biotic interactions can constrain the areas in which a species can actually persist. We attempted to include the availability of suitable habitat in our models by creating a GIS layer that estimates the amount of wetlands available across the contiguous US and Ontario. Therefore, the areas predicted suitable by our model should only be considered truly at risk from invasion if the water bodies present possess suitable habitat (e.g., emergent vegetation) and prey throughout the active season of these snakes. Even though we did not include any biological variables in our environmental predictors, there is reason to believe that much of the area that is climatically suitable for *N. fasciata* and *N. sipedon* in western North America is also biotically suitable for these species. River systems in western states have been dammed, channelized, and modified, changing flow regimes to be more similar to other regions of North America [64] and facilitating establishment of invasive species at the expense of natives [12], [13]. Several fish and amphibian species that co-occur with *Nerodia* in eastern North America are already well-established in the west, including the American Bullfrog, *Lithobates catesbeianus* [65], and a variety of species in the Sunfish family (Centrarchidae) [66]. Indeed, since European colonization, California's fish fauna has become more similar to that of the eastern US due to the introduction of fish native to eastern states into California [67]. Previous invasion by co-occurring species can facilitate the invasion of non-native species [68], and both *N. fasciata* and *N. sipedon* feed on species already established in California, such as Sunfish and American Bullfrogs, in their native range [28].

Another factor in determining the invasion risk of non-native watersnakes is the ability of individuals to spread from established populations and colonize suitable but unoccupied habitats. The extensive canal systems for agriculture and water diversion in California's Central Valley create a highly networked system of suitable aquatic habitats that could greatly facilitate the spread of *Nerodia* across the state. This is borne out in the results of our connectivity analysis, which showed that for both watersnake species the vast majority of suitable habitat in the Central Valley is directly connected to established populations. The home ranges of both *N. fasciata* and *N. sipedon* can cover several hectares and multiple water bodies [69], [70], *N. sipedon* individuals can move more than 500 m along a stream corridor in a single day [71], and *N. fasciata* may travel long distances through terrestrial habitat in response to changing hydrological conditions [72]. In addition, humans may directly facilitate the spread of non-native watersnakes by capturing them in one location to serve as a pet, and later releasing them elsewhere. Indeed, the most parsimonious explanation for the establishment of the three currently known, distinct *Nerodia* populations in California is that each represents an independent release of captive animals. All of these factors make it likely that *N. fasciata* and *N. sipedon* will find hospitable conditions in freshwater habitats in California that allow them to persist and spread beyond their current locations if they are not eradicated in this early stage of establishment.

The methods we used in this study provide a systematic, quantitative, spatial method for evaluating threats posed to native species by invaders. We used knowledge of the natural history of the invaders, including diet and habitat preferences, to identify native species likely to compete with or be prey for *Nerodia*. For example, the threatened Giant Gartersnake, *T. gigas*, is known to occupy a similar ecological niche to snakes in the genus *Nerodia*, being restricted to aquatic habitats where it forages for fish and amphibians [73]. Other species were chosen because *N. fasciata* or *N. sipedon* feed on these species in their native range (e.g., *O. mykiss*) or feed on closely related members of the same genus or family (e.g., *A. californiense*, *R. draytonii*). We converted our continuous ensemble model output to a binary presence/absence prediction using a threshold (Max SSS) that balances the probability of predicting both false negatives, which may be costly when modeling the range of invasive species because they can lead to underestimates of invasion risk [36], and false positives, which would overstate the risk posed by an invader. We then quantified the predicted degree of overlap between these species' native ranges and the potential invasive ranges of *N. fasciata* and *N. sipedon*. This method identifies the degree to which several of California's imperiled species are likely to come into conflict with *Nerodia* and where these conflicts are likely to arise. Many previous studies have used ecological niche modeling to predict the risk of spread of invasive species [23], [37], but few take the next step to connect this risk to the presence of threatened native species. Phillips et al. [74] quantified the risk posed by the Cane Toad, *Rhinella marina*, to native snakes in Australia based on potential range overlap, but based their conclusions on only one species distribution model, thereby failing to account for uncertainty in projecting the potential distribution of a rapidly spreading invader. Our method provides a template that could be employed by government agencies to evaluate the risk posed by other introduced species. Additionally, while we focused our analysis on native imperiled species, these models could be applied to examine risk for currently widespread or common native fauna to anticipate unforeseen conflicts or possible native species declines from invasives before they occur.

Despite their excellent predictive performance according to AUC, our ensemble models do demonstrate some systematic errors. For example the models overestimate the native range of both *Nerodia* species, extending the range of *N. fasciata* north of the Gulf coastal plain in Alabama and Mississippi and *N. sipedon* into areas west of its known range. This over-prediction is likely due to our method of pseudo-absence selection. Because we created models using museum records and haphazardly collected observations, we had no true absence data and had to generate pseudo-absences outside the known range of these species. To prevent the model from under-predicting the suitable area for these species, we only selected pseudo-absences that are at a minimum 2 degrees distance from known occurrences. This prevents the SDMs from training on any data in the intervening area between the presence localities and pseudo-absences, and due to the climatic similarity between adjacent occupied and unoccupied areas, the models predict the area as suitable. An interesting question, but one outside the focus of this study, is whether some of these areas are in fact suitable for one species in the absence of the other. For example, is the Gulf Coast of South Carolina suitable for *N. sipedon* climatically, with only competition from *N. fasciata* preventing it from expanding its range into this region? The ensemble model for *N. sipedon* also slightly under-predicts its native range, with low suitability in eastern Minnesota and western Wisconsin. This under-prediction is likely due to a paucity of occurrence records from this region, likely combined with the cold temperatures that are on the edge of suitable climate for *N. sipedon*.

Our results suggest that if *N. fasciata* and *N. sipedon* are allowed to spread throughout California, they may represent another significant stressor to many native aquatic species that are already imperiled. The Common Watersnake, *N. sipedon*, may pose the greater threat as an invasive species because it has the potential to spread farther north due to its broad native distribution and presumably greater climatic tolerance. In contrast, the Southern Watersnake, *N. fasciata*, has a more restricted climatic niche, but may still spread throughout the Central Valley of California, where native amphibians and fish have already suffered significant declines [14], [75]. Perhaps more alarmingly, these two watersnake species frequently interbreed in areas of sympatry along the edge of their native ranges, specifically in ecotones and environmentally disturbed areas [76]–[79]. Hybridization between two species or genetically distinct populations can increase their invasiveness due to increased genetic variation and the generation of novel genotypes [80]. Thus, it is possible that these two watersnake species, with established populations in California currently located only 20 km apart, could interbreed and produce hybrid genotypes with broader climatic tolerances, making them even better suited to the array of climates represented across California and the western US. We recommend that urgent action be taken now to control emergent populations of these non-native snakes while they presumably remain somewhat restricted in California. Finally, our methods could be adapted and used by biologists studying incipient invasions in other systems to quantify the potential risk to imperiled native species posed by introduced species.

## Supporting Information

Figure S1 **Response curves from a Boosted Regression Tree species distribution model for** ***N. fasciata*** **.** Y-axes are on the logit scale.
(TIF)

Figure S2 **Response curves from a Maxent species distribution model for** ***N. fasciata*** **.**
(TIF)

Figure S3 **Response curves from a Random Forest species distribution model for** ***N. fasciata*** **.**
(TIF)

Figure S4 **Response curves from a Boosted Regression Tree species distribution model for** ***N. sipedon*** **.** Y-axes are on the logit scale.
(TIF)

Figure S5 **Response curves from a Maxent species distribution model for** ***N. sipedon*** **.**
(TIF)

Figure S6 **Response curves from a Random Forest species distribution model for** ***N. sipedon*** **.**
(TIF)

Appendix S1 **Expanded modeling methods and model evaluation.**
(DOCX)

Table S1 **Native species potentially threatened by invasive** ***Nerodia*** **and their conservation status.**
(DOCX)

Table S2 **AUC values for spatially stratified cross-validation for** ***Nerodia fasciata*** **.**
(DOCX)

Table S3 **AUC values for spatially stratified cross-validation for** ***Nerodia sipedon*** **.**
(DOCX)
