# Supplementary figures and images for: Projecting Invasion Risk of Non-Native Watersnakes (Nerodia fasciata and Nerodia sipedon) in the Western United States
Source: PLoS One. 2014 Jun 25;9(6):e100277. doi: 10.1371/journal.pone.0100277 (PMC4070932; doi:10.1371/journal.pone.0100277)

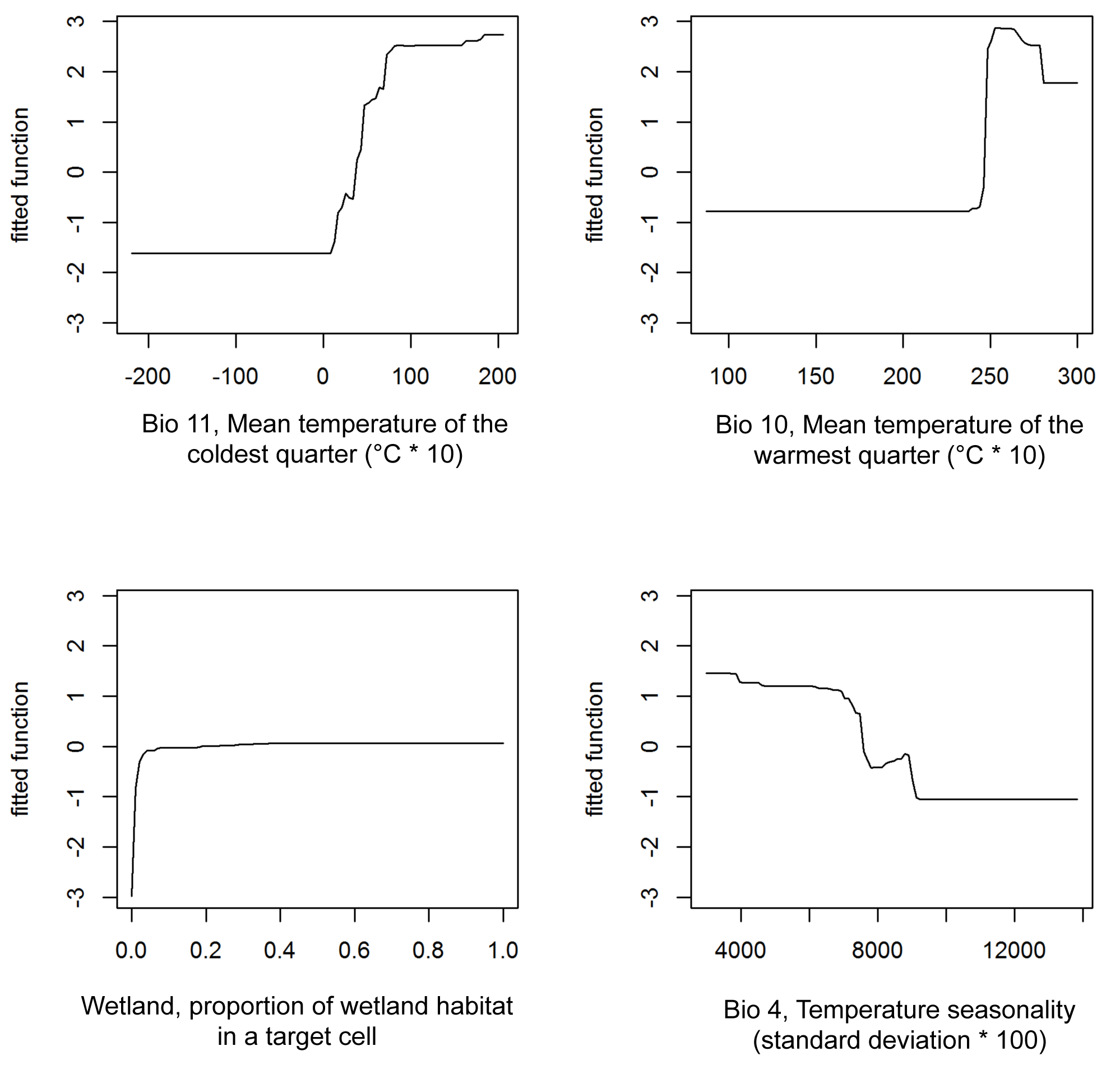

Supplement: Figure S1 — Response curves from a Boosted Regression Tree species distribution model for N. fasciata . Y-axes are on the logit scale. (TIF) [file pone.0100277.s001.tif]

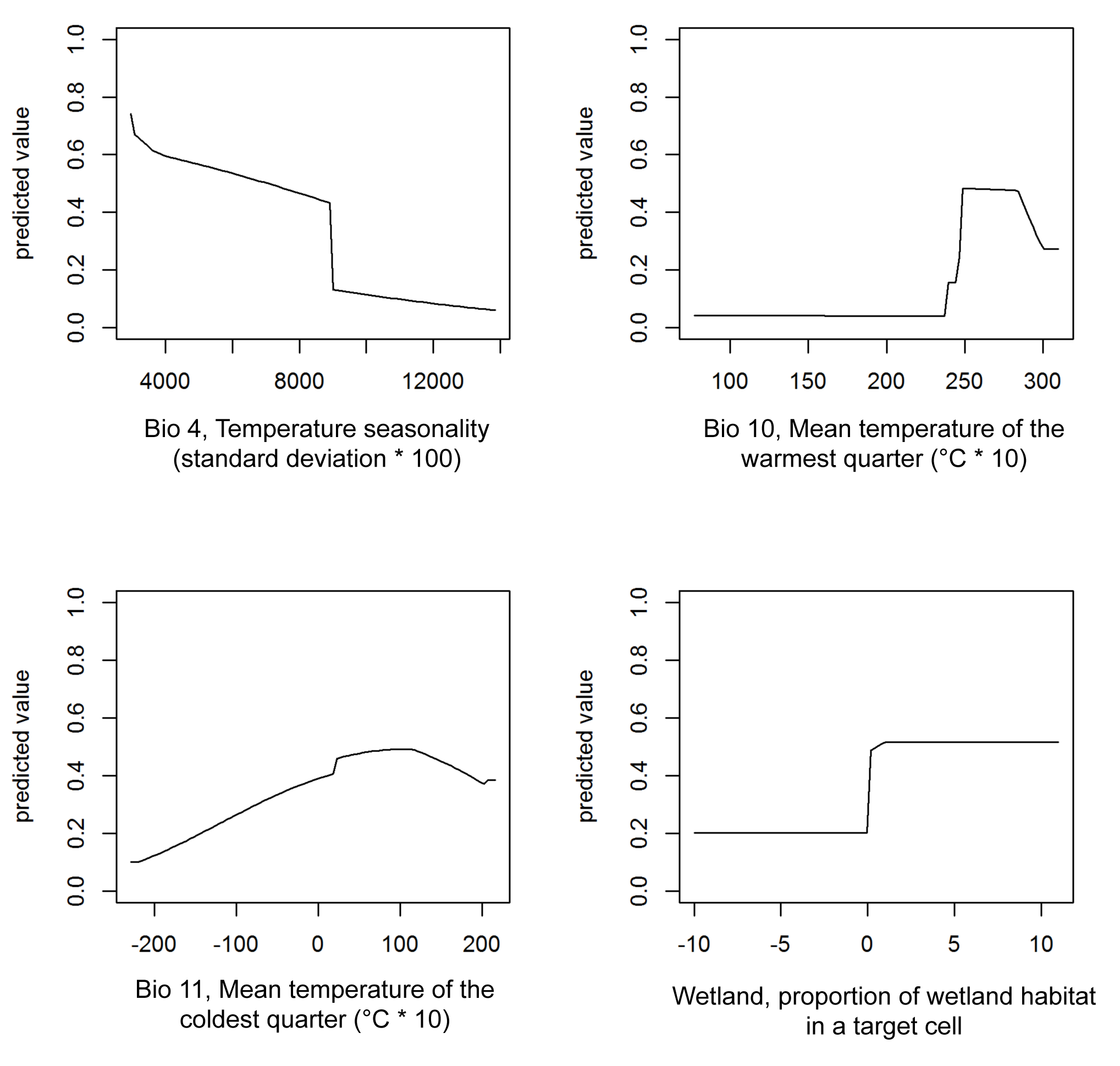

Supplement: Figure S2 — Response curves from a Maxent species distribution model for N. fasciata . (TIF) [file pone.0100277.s002.tif]

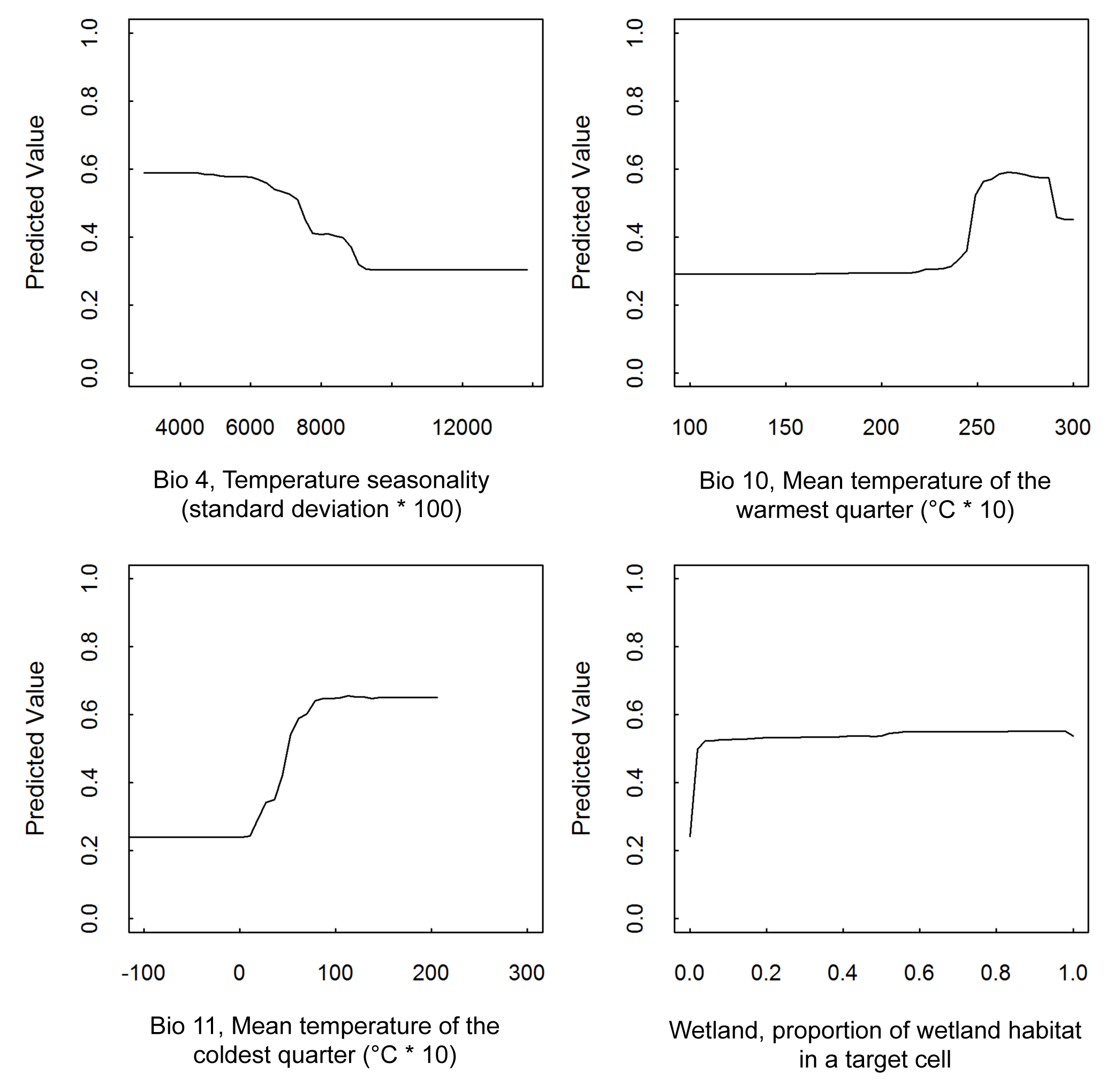

Supplement: Figure S3 — Response curves from a Random Forest species distribution model for N. fasciata . (TIF) [file pone.0100277.s003.tif]

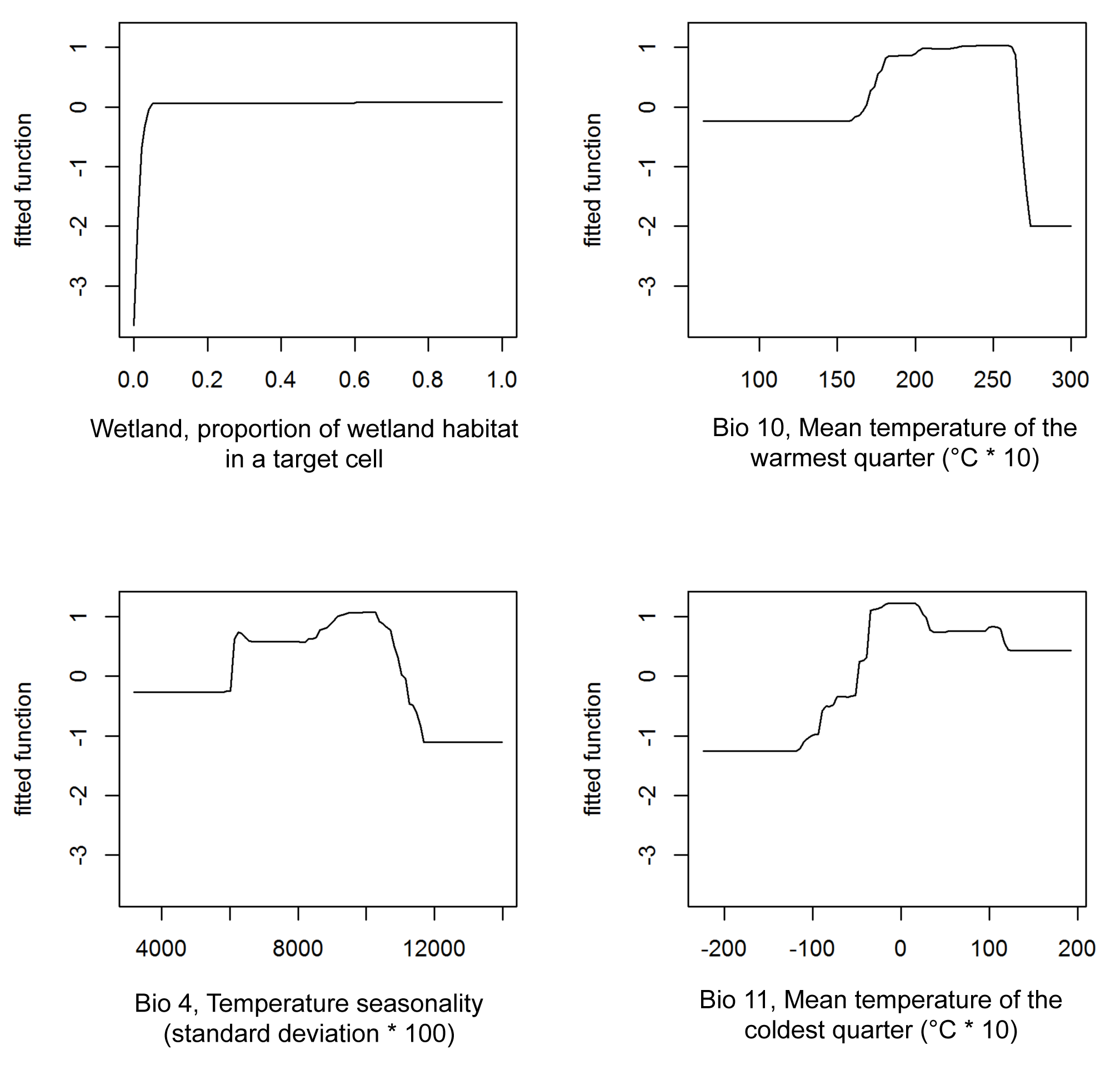

Supplement: Figure S4 — Response curves from a Boosted Regression Tree species distribution model for N. sipedon . Y-axes are on the logit scale. (TIF) [file pone.0100277.s004.tif]

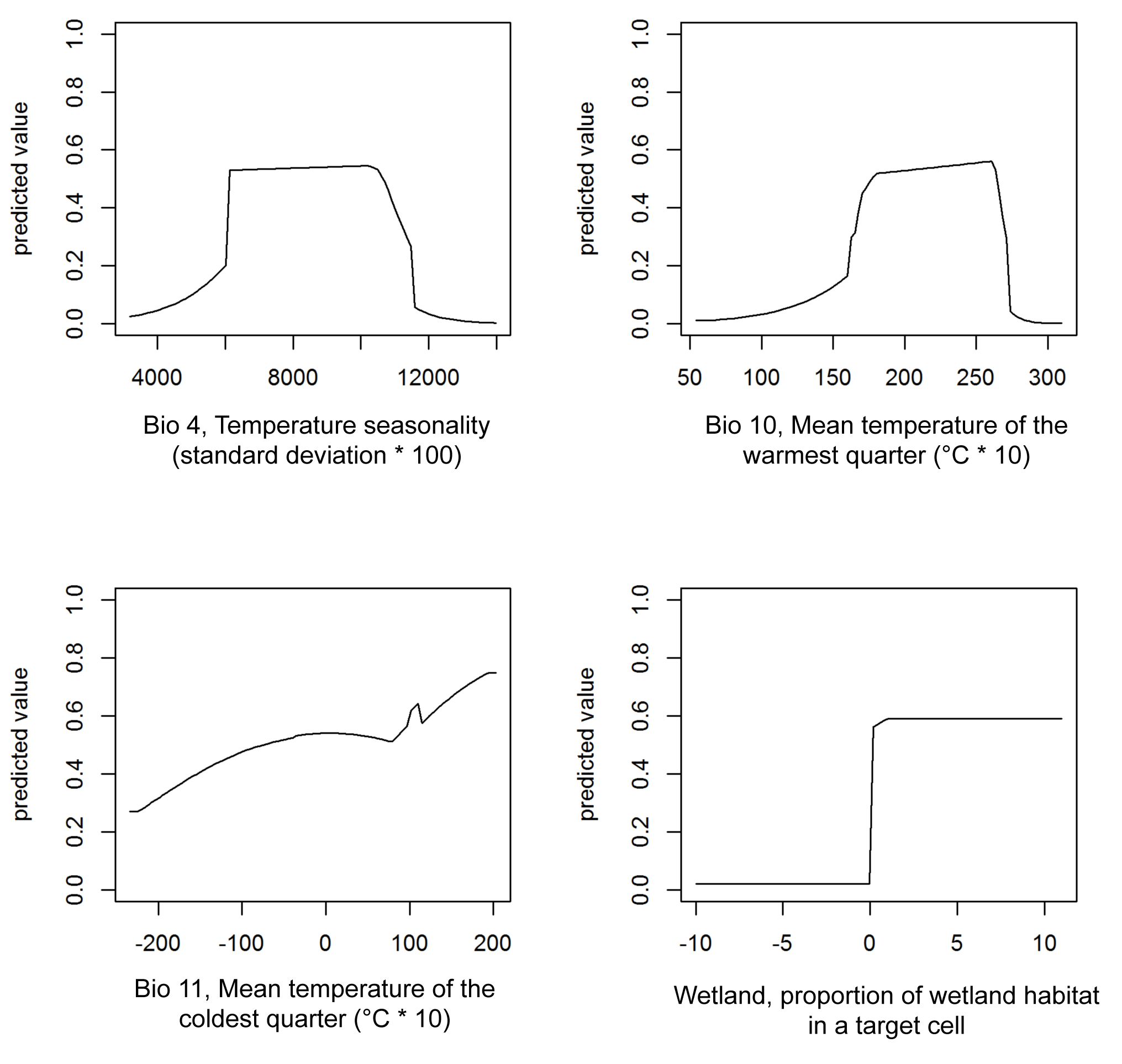

Supplement: Figure S5 — Response curves from a Maxent species distribution model for N. sipedon . (TIF) [file pone.0100277.s005.tif]

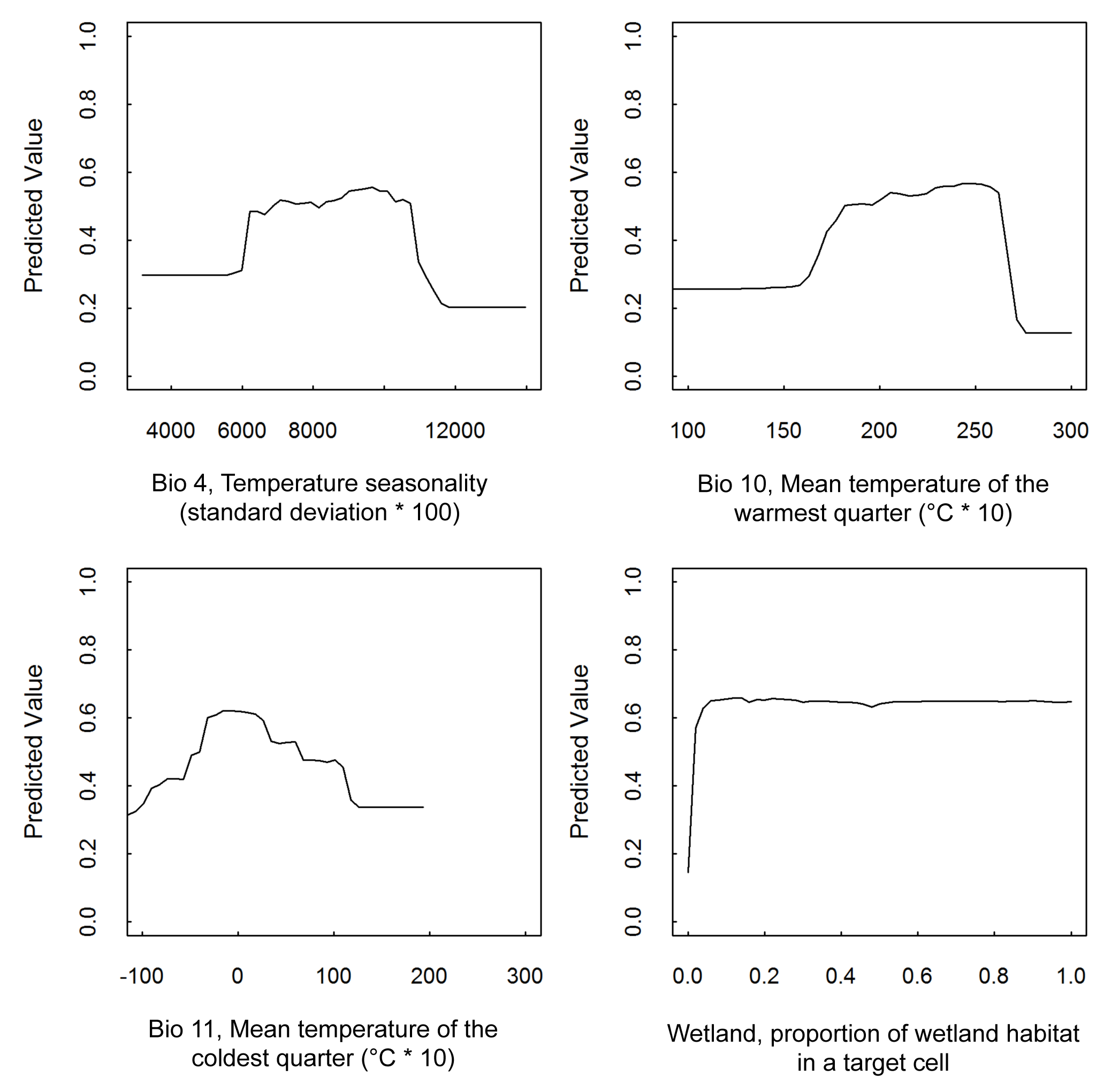

Supplement: Figure S6 — Response curves from a Random Forest species distribution model for N. sipedon . (TIF) [file pone.0100277.s006.tif]
