# Supplementary material for: Projecting Invasion Risk of Non-Native Watersnakes (Nerodia fasciata and Nerodia sipedon) in the Western United States
Source: PLoS One. 2014 Jun 25;9(6):e100277. doi: 10.1371/journal.pone.0100277 (PMC4070932; doi:10.1371/journal.pone.0100277)
Supplement: Appendix S1 — Expanded modeling methods and model evaluation. (DOCX) [file pone.0100277.s007.docx]

Appendix S1 – Expanded modeling methods and model evaluation.

***Pseudo-absence generation***

Boosted regression trees and Random forests both require absence data to contrast with known occurrences, while Maxent requires “background” points both from areas where the species is present and where it is absent. An important decision in presence-only modeling is how to delineate the geographic background against which environmental characteristics of species occurrences will be compared. The choice of background can greatly influence model results [1,2], and ideally the background should represent the geographic area that has been “accessible” to the species in question (i.e., not beyond a dispersal barrier such as a major river or mountain range; Peterson et al. 2011). One way to define this background is to select all ecoregions within which a species occurs [2]. To define the geographic area accessible to *N. sipedon* and *N. fasciata*, we selected all provinces from Bailey’s ecoregions of North America [3] in which the genus *Nerodia* occurs. This captures the area accessible to *N. sipedon* and *N. fasciata* because if other species in the genus *Nerodia* (whose ranges overlap with *N. fasciata* and *N. sipedon*) occur in a region, it is likely that there is no dispersal barrier preventing our target species from reaching that area, and thus they are likely absent due to unsuitable habitat or climate. We also selected an additional ecoregion to the north (Ecoregion 232: Taiga-boreal forest) in which no *Nerodia* occur, but this absence is almost certainly due to the cold climate in the ecoregion and not to the presence of a dispersal barrier. We did not use occurrences of *N. clarkii* in our background selection; this species inhabits brackish and saltwater habitats and its range extends south to Cuba. Because saltwater likely presents a dispersal barrier to *N. sipedon* and *N. fasciata* (except for the narrowly distributed *N.s. williamengelsi*), we excluded Cuba from our background. This background was further reduced to only include one Canadian province, Ontario, for which wetlands data were available.

Pseudo-absences were randomly generated within the background area described above but with a minimum distance of 2^o^ from known presence points following [4]. For Maxent, the presence points were combined with the randomly generated pseudo-absence points to create the background points.

***Modeling methods***

Below we describe the details of our parameter selections for each of the three modeling methods and the rationale behind each choice.

*Boosted Regression Trees*

All trees were built using a binomial response type using a logit link. The bag fraction was set to 0.5, so 50% of the data was randomly drawn and used to fit each individual tree following Elith et al. [5]. This random data selection can decrease over-fitting and increase model accuracy [6]. We chose the values for learning rate, tree complexity, and number of trees by building models with varying values for learning rate and tree complexity, and selecting those values that minimized the predictive deviance and maximized AUC on latitudinal bands that were withheld from model training. We set tree complexity to three, allowing up to three-way interactions between our environmental predictor variables. We set the learning rate to 0.005 and the number of trees to 1,000 because these values were found to minimize the deviance remaining on withheld data on average (specifically the northern and southernmost latitudinal bands), while preventing the fitted response functions (Figures S1, S4) from becoming overly complex. Elith et al. [7] found that BRT performed better at extrapolating to novel climates when fitted functions were constrained to be smooth by limiting the number of trees in the model. Therefore, by limiting the number of trees in our BRT models, we prevented the model from becoming over-fit to the training data and increased its ability to transfer to regions in which it was not trained. All BRT models were built using the “gbm” package in R version 2.15.3 [8]

*Maxent*

We ran all Maxent models with automatic feature selection and the model set to clamp values when projecting beyond the range of the training data to the maximum value in the training data set. We followed the methods of Warren and Seifert [9] to select the optimal value of beta for model regularization. We calculated AICc for all Maxent models when predicting to withheld latitudinal bands while varying the value of β from 0.5 - 4. Larger values of beta lead to smoother species response curves and can improve predictions when extrapolating to novel climate spaces [7]. Our results for choosing β varied depending on which band was held back for model evaluation, but overall based on AICc, we found the best value of β to be 1 for *N. fasciata* and 1.1 for *N. sipedon*. While the response curves for these models do show some sharp thresholds in suitability, they appear biologically intuitive and show little evidence of over-fitting (Figure S2, S5). All Maxent models were built using the “dismo” package in R version 2.15.3 [8].

*Random Forests*

We set all models to build 500 regression trees and use only one variable at each split, as the standard is to use one third of the total predictors available at each split [10]. To prevent over-fitting, we built several models with varying values for node-size, which determines the minimum number of records at each terminal node and therefore effectively limits the number of nodes in each tree. Following the same procedure for BRT above, we evaluated which values for node-size minimized the predictive deviance and maximized AUC for withheld latitudinal bands. We also evaluated response curves (Figure S3, S6) from models with different values for node-size for evidence of over-fitting. Based on our model calibration, we ran our final models with a node-size of 50 for *N. fasciata* and 15 for *N. sipedon*. We measured variable importance by calculating the change in prediction accuracy (as measured by mean-squared error) on out-of-bag data when each predictor variable is randomly permuted [11]. All RF models were built with the “randomForest” package in R version 2.15.3 [8].

***Spatially stratified validation***

To evaluate our models’ ability to extrapolate beyond its training data, we used a spatially stratified validation method [12,13] where we divided each species’ range into five latitudinal bands, with bands each containing one-fifth of the species’ total presences. We then built a model using occurrences and pseudo-absences from four bands, while withholding one band for testing model performance. We repeated this process five times until each subset had been used once for testing. From these models we generated AUC values for each latitudinal band and calculated the mean AUC for each model over all bands to test how well each model is able to transfer its predictive ability beyond the training area. We chose latitudinal bands because temperature varies much more with latitude than longitude, and therefore withholding latitudinal bands requires our models to project into novel environmental space to a greater degree than if we had used longitudinal bands. Thus, this simulates the ultimate application of our models, projecting into the novel environmental space in western North America, and provides a strong test of our models’ transferability [14]. As stated in our methods, our use of AUC is justified because all models were defined by the same background area, and we do not compare AUC between SDMs for different species, we only compare AUC between models that differ in the machine learning method used. The results of our spatially-stratified validation are presented in Tables S2 and S3. The best performing model varied by latitude, with RF having the highest AUC for 3/5 bands for *N. fasciata* and for *N. sipedon*.

***Native species distributions***

For reptiles and amphibians, we used range maps obtained either from the California Wildlife Habitat Relationships program for California endemics [15] or from IUCN for species with ranges that extend beyond California. For California fish species, we used data from CDFG’s CalFish program for salmonids (<http://www.calfish.org/DataandMaps/CalFishDataDownloads/tabid/93/Default.aspx>) and California range maps from Moyle & Randall [15]; available at <http://ice.ucdavis.edu/aquadiv/fishcovs/fishmaps.html>) for all other species. We used the extract by mask tool in ArcMap version 9.3 to clip our ensemble models by each native species’ range, to estimate the suitability of the native species’ range for the introduced *Nerodia*.

***Wetlands layer***

To download the wetlands raster layer we created from the USGS National Hydrography Dataset and the Canadian Wetlands Inventory data from the Ontario Ministry of Natural Resources, please visit our lab website at (http://toddlab.ucdavis.edu/nerodia.html).

References

1. Anderson RP, Raza A (2010) The effect of the extent of the study region on GIS models of species geographic distributions and estimates of niche evolution: preliminary tests with montane rodents (genus Nephelomys) in Venezuela. J Biogeogr 37: 1378–1393.

2. Barve N, Barve V, Jiménez-Valverde A, Lira-Noriega A, Maher SP, et al. (2011) The crucial role of the accessible area in ecological niche modeling and species distribution modeling. Ecol Modell 222: 1810–1819.

3. Bailey RG (1997) Map: Ecoregions of North America (revised). Washington, D.C.: USDA Forest Service in cooperation with the Nature Conservancy and the US Geological Survey.

4. Barbet-Massin M, Jiguet F, Albert CH, Thuiller W (2012) Selecting pseudo-absences for species distribution models: how, where and how many? Methods Ecol Evol 3: 327–338.

5. Elith J, Leathwick JR, Hastie T (2008) A working guide to boosted regression trees - Online Appendices Page 1. J Anim Ecol: 1–4.

6. Friedman JH (2002) Stochastic gradient boosting. Comput Stat Data Anal 38: 367–378.

7. Elith J, Kearney M, Phillips S (2010) The art of modelling range-shifting species. Methods Ecol Evol 1: 330–342.

8. R: A language and environment for statistical computing. (2013).

9. Warren DL, Seifert SN (2011) Ecological niche modeling in Maxent: the importance of model complexity and the performance of model selection criteria. Ecol Appl 21: 335–342.

10. Prasad AM, Iverson LR, Liaw A (2006) Newer Classification and Regression Tree Techniques: Bagging and Random Forests for Ecological Prediction. Ecosystems 9: 181–199.

11. Cutler DR, Edwards TC, Beard KH, Cutler A, Hess KT, et al. (2007) Random forests for classification in ecology. Ecology 88: 2783–2792.

12. Jiménez-Valverde A, Peterson A, Soberón J, Overton JM, Aragón P, et al. (2011) Use of niche models in invasive species risk assessments. Biol Invasions 13: 2785–2797.

13. Peterson AT, Soberón JM, Pearson RG, Anderson RP, Martinez-Meyer E, et al. (2011) Ecological Niches and Geographic Distributions. Princeton: Princeton University Press.

14. Wenger SJ, Olden JD (2012) Assessing transferability of ecological models: an underappreciated aspect of statistical validation. Methods Ecol Evol 3: 260–267.

15. California Department of Fish & Game (2008) California Wildlife Habitat Relationships.

16. Moyle PB, Randall PJ (1998) Evaluating the Biotic Integrity of Watersheds in the Sierra Nevada, California. Conserv Biol 12: 1318–1326.
