# Supplementary material for: Projecting Invasion Risk of Non-Native Watersnakes (Nerodia fasciata and Nerodia sipedon) in the Western United States
Source: PLoS One. 2014 Jun 25;9(6):e100277. doi: 10.1371/journal.pone.0100277 (PMC4070932; doi:10.1371/journal.pone.0100277)
Supplement: Table S1 — Native species potentially threatened by invasive Nerodia and their conservation status. (DOCX) [file pone.0100277.s008.docx]

Table S1. Native species potentially threatened by invasive *Nerodia* and their conservation status.

| Species | Threat Status |
| --- | --- |
| Amphibians |  |
| *Ambystoma californiense* | USFWS-T,E* |
| *Ambystoma macrodactylum croceum* | USFWS-E |
| *Ambystoma macrodactylum sigillatum* | CDFW-SSC |
| *Ascaphus truei* | CDFW-SSC |
| *Dicamptodon ensatus* | CDFW-SSC |
| *Rana boylii* | CDFW-SSC |
| *Rana cascadae* | CDFW-SSC |
| *Rana draytonii* | USFWS-T |
| *Rana muscosa* | USFWS-E |
| *Rana sierrae* | USFWS-C |
|  |  |
| Fish |  |
| Klamath Mountains summer steelhead | CDFW-SSC |
| Southern California steelhead | USFWS-E |
| Northern California coast summer steelhead | USFWS-T |
| Central Valley steelhead | USFWS-T |
| South Central California coast steelhead | USFWS-T |
| Central California coast steelhead | USFWS-T |
| Northern California coast winter steelhead | Not listed |
| Klamath Mountains winter steelhead | Not listed |
| *Deltistes luxatus* | USFWS-E |
| *Gila orcutti* | CDFW-SSC |
| *Hypomesus transpacificus* | USFWS-T |
| *Lampetra hubbsi* | CDFW-SSC |
| *Lavinia exilicauda* | CDFW-SSC** |
| *Pogonichthys macrolepidotus* | CDFW-SSC |
| *Lavinia symmetricus* | CDFW-E, SSC*** |
| *Hysterocarpus traski* | CDFW-SSC**** |
|  |  |
| Reptiles |  |
| *Thamnophis atratus* | LC |
| *Thamnophis couchii* | LC |
| *Thamnophis elegans* | LC |
| *Thamnophis gigas* | USFWS-T |
| *Thamnophis hammondii* | CDFW-SSC |
| *Thamnophis ordinoides* | LC |
| *Thamnophis sirtalis tetrataenia* | USFWS-E |

*Two distinct population segments of *Ambystoma californiense* are listed as endangered, whereas the central valley DPS is listed as threatened. **Clear lake subspecies only. ***The Red hills roach is listed as endangered, whereas the Pit roach is a species of special concern. ****Russian river subspecies only. For our analyses these populations were pooled. Status codes: LC = Least Concern by IUCN, CDFW-SSC = Species of Special Concern by the California Dept. of Fish and Wildlife, USFWS-E = Listed as endangered under the federal Endangered Species Act, USFWS-T = Listed as threatened under the federal ESA, USFWS-C = Listed as a candidate for listing under the federal ESA.
