# Supplementary material for: Projecting Invasion Risk of Non-Native Watersnakes (Nerodia fasciata and Nerodia sipedon) in the Western United States
Source: PLoS One. 2014 Jun 25;9(6):e100277. doi: 10.1371/journal.pone.0100277 (PMC4070932; doi:10.1371/journal.pone.0100277)
Supplement: Table S2 — AUC values for spatially stratified cross-validation for Nerodia fasciata . (DOCX) [file pone.0100277.s009.docx]

Table S2. AUC values for spatially-stratified cross-validation by state for *Nerodia fasciata.*

| *Nerodia fasciata* | | Model |  |
| --- | --- | --- | --- |
| Latitudinal Band | BRT | Maxent | RF |
| 1 | 0.976 | 0.959 | 0.976 |
| 2 | 0.993 | 0.997 | 0.999 |
| 3 | 0.989 | 0.994 | 0.986 |
| 4 | 0.982 | 0.988 | 0.993 |
| 5 | 0.929 | 0.983 | 0.932 |
