# Supplementary material for: Projecting Invasion Risk of Non-Native Watersnakes (Nerodia fasciata and Nerodia sipedon) in the Western United States
Source: PLoS One. 2014 Jun 25;9(6):e100277. doi: 10.1371/journal.pone.0100277 (PMC4070932; doi:10.1371/journal.pone.0100277)
Supplement: Table S3 — AUC values for spatially stratified cross-validation for Nerodia sipedon . (DOCX) [file pone.0100277.s010.docx]

Table S3. AUC values for spatially-stratified cross-validation by state for *Nerodia sipedon.*

| *Nerodia sipedon* | | Model |  |
| --- | --- | --- | --- |
| Latitudinal Band | BRT | Maxent | RF |
| 1 | 0.927 | 0.939 | 0.950 |
| 2 | 0.893 | 0.906 | 0.887 |
| 3 | 0.991 | 0.996 | 0.999 |
| 4 | 0.990 | 0.990 | 0.999 |
| 5 | 0.865 | 0.902 | 0.882 |
